# Discrimination learning and judgment bias in low birth weight pigs

**DOI:** 10.1007/s10071-019-01262-5

**Published:** 2019-05-03

**Authors:** Sanne Roelofs, Floor A. C. Alferink, Allyson F. Ipema, Tessa van de Pas, Franz Josef van der Staay, Rebecca E. Nordquist

**Affiliations:** 1 Behaviour and Welfare Group, Department of Farm Animal Health, Faculty of Veterinary Medicine, Utrecht University, Yalelaan 7, 3584 CL Utrecht, The Netherlands; 2 Brain Center Rudolf Magnus, Utrecht University, Stratenum Building, Room STR5.203, Universiteitsweg 100, 3584 CG Utrecht, The Netherlands; 3 Present Address: Department of Clinical Studies, Swine Teaching and Research Center, New Bolton Center, School of Veterinary Medicine, University of Pennsylvania, 382 W Street Road, Kennett Square, PA 19348 USA; 4 Study Programme Applied Biology, HAS University of Applied Sciences, Onderwijsboulevard 221 5223 DE ‘s Hertogenbosch, The Netherlands; 5 Adaptation Physiology Group, Wageningen University, De Elst 1, 6708 WD Wageningen, The Netherlands

**Keywords:** Pigs, Birth weight, Cognition, Emotion, Cognitive bias, Ambiguity

## Abstract

Low birth weight (LBW) is a risk factor for cognitive and emotional impairments in humans. In pigs, LBW is a common occurrence, but its effects on cognition and emotion have received only limited scientific attention. To assess whether LBW pigs suffer from impaired cognitive and emotional development, we trained and tested 21 LBW and 21 normal birth weight (NBW) pigs in a judgment bias task. Judgment bias is a measure of emotional state which reflects the influence of emotion on an animal’s interpretation of ambiguous stimuli. Pigs were trained to perform a specific behavioral response to two auditory stimuli, predicting either a positive or negative outcome. Once pigs successfully discriminated between these stimuli, they were presented with intermediate, ambiguous stimuli. The pigs’ responses to ambiguous stimuli were scored as optimistic (performance of ‘positive’ response) or pessimistic (performance of ‘negative’ response). Optimistic or pessimistic interpretation of an ambiguous stimulus is indicative of a positive or negative emotional state, respectively. We found LBW pigs to require more discrimination training sessions than NBW pigs to reach criterion performance, suggesting that LBW causes a mild cognitive impairment in pigs. No effects of LBW on judgment bias were found, suggesting a similar emotional state for LBW and NBW pigs. This was supported by comparable salivary and hair cortisol concentrations for both groups. It is possible the enriched housing conditions and social grouping applied during our study influenced these results.

## Introduction

Low birth weight (LBW) is a known risk-factor for impaired cognitive and emotional development in humans. Children who were small for gestational age at birth are more likely to experience learning difficulties (O’Keeffe et al. 2003; Yu and Garcy 2018) and show impaired academic performance (Strauss 2000; Larroque et al. 2001; Lindström et al. 2017) throughout childhood and adolescence. In terms of emotional development, lower birth weight is associated with increased likelihood of anxiety in adulthood (Lahti et al. 2010). Furthermore, being small for gestational age increases the risk of developing emotional disorders (e.g., anxiety disorder, depression) in preterm babies (Boyle et al. 2011; Lahat et al. 2017). Together, these studies show that LBW can have long-lasting effects on the highly associated processes of cognitive and emotional functioning (Lazarus 1982).

LBW is also becoming a common occurrence in commercially housed pigs. This is a result of sows producing increasingly large litters (Rutherford et al. 2013) and being unable to provide sufficient oxygen and nutrients for proper development of all of the fetuses (Père and Etienne 2000; Wähner and Fischer 2005). The resulting intra-uterine growth restriction is very comparable to how LBW develops in humans (Cox and Marton 2009; Gayatri et al. 2017). Unlike in humans, however, the long-term effects of LBW on cognition and emotion have not yet been extensively studied in pigs. Such potential effects are of interest, as pigs depend on both learning and memory, as well as emotional processes, for successful coping within their environment (Held et al. 2002; Boissy et al. 2007).

The effects of LBW on post-weaning cognitive performance in pigs are not fully understood, as prior studies have produced contradictory results. For example, LBW pigs have been reported to show impaired (Gieling et al. 2012; Radlowski et al. 2014; Roelofs et al. 2018), similar (Gieling et al. 2014) or even improved (Antonides et al. 2015) spatial learning and memory compared to normal birth weight (NBW) pigs. Studies aimed at the post-weaning emotional functioning of LBW pigs have mostly relied on physiological measures of stress, mainly plasma cortisol concentration (Rutherford et al. 2013). Results of these studies suggest that LBW pigs react more strongly to acute stressors than NBW pigs (e.g., Poore et al. 2002; Poore and Fowden 2003). Behavioral measures of emotional state in pigs, which can provide a better indication of the valence of experienced emotions (Murphy et al. 2014), have not yet been widely applied to compare LBW and NBW pigs.

Judgment bias is one such behavioral measure of emotional state in animals. It describes the influence of emotion on the interpretation of ambiguous, i.e., emotionally neutral, stimuli (Paul et al. 2005; Mendl et al. 2009). To measure judgment bias, animals are first trained to successfully discriminate between a stimulus signaling a positive outcome (e.g., a food reward) and a stimulus signaling a negative outcome (e.g., punishment or a smaller food reward). The animal performs a different behavioral response to each stimulus, and these responses are then used to assess judgment bias. If an animal performs the behavior it has learned to associate with a positive outcome after being presented with an ambiguous stimulus (often intermediate between the positive and negative stimuli), this is scored as an optimistic response. If it performs the behavior it has learned to associate with a negative outcome, this is scored as a pessimistic response. An animal which makes more optimistic responses is assumed to be in a more positive emotional state than an animal which makes more pessimistic responses (Mendl et al. 2009; Roelofs et al. 2016). Judgment bias tasks have been successfully applied to a variety of species, including humans and pigs (Miranda and Mennin 2007; Roelofs et al. 2016). For example, pigs respond more optimistically to ambiguous stimuli when housed in enriched conditions which were assumed to improve their emotional state (Douglas et al. 2012). Because discrimination training is a necessary component of a judgment bias task, it allows for successive assessment of discrimination learning and emotional state (i.e., judgment bias).

Murphy and colleagues have previously compared LBW and NBW pigs in a judgment bias task (Murphy et al. 2015). They found LBW pigs to be equally capable of mastering the conditional discrimination task, but LBW pigs displayed a more negative judgment bias than NBW pigs. However, in their study only male pigs were tested. Accounting for a potential difference between females and males is relevant when training and testing LBW pigs in a judgment bias task. First, LBW pigs have been found to display altered stress responses compared to NBW pigs (Poore and Fowden 2003). Stress is known to influence learning and memory abilities, and such effects can be sex-specific (Bowman et al. 2003; Healy et al. 2009). Second, the increased risk of emotional disorders due to LBW found in humans appears to affect females more than males (Costello et al. 2007; Van Lieshout and Boylan 2010). Therefore, repeating a judgment bias study with both female and male LBW pigs is relevant for assessing the potential differential effect of sex on the performance of LBW in pigs.

The aim of the present study was to assess the effects of birth weight on discrimination learning and judgment bias in pigs. These effects were assessed by a judgment bias task, where the pigs chose a goal location based on a positive and a negative stimulus. Several improvements to a previous study assessing judgment bias in LBW pigs were applied (Murphy et al. 2015). First, we assessed a larger sample size consisting of both female and male pigs to account for possible differential effects of stress on LBW pigs’ cognition and emotional state. Second, to further assess a potential difference in stress response between LBW and NBW pigs, markers of acute and chronic stress (salivary and hair cortisol concentrations, respectively) were included. We expected LBW pigs would show impaired discrimination learning and a more pessimistic judgment bias compared to NBW pigs. Additionally, LBW pigs were expected to have an exaggerated stress response, with increased hair cortisol concentrations and a stronger salivary cortisol response to an acute stressor.

## Materials and methods

### Animals

Pigs [(Yorkshire × Dutch Landrace) × Duroc] were selected from the commercial pig breeding farm of Utrecht University. From 14 different litters, 21 LBW–NBW sibling pairs were selected (11 female pairs and ten male pairs). Eight litters provided a single sibling pair, five litters provided two sibling pairs and one litter provided three sibling pairs. The experiment took place in two separate rounds due to limited availability of LBW piglets, with 20 piglets selected for the first round (10 LBW–NBW pairs, trained and tested in March–June 2017) and 22 piglets selected for the second round (11 LBW–NBW pairs, trained and tested in August–November 2017). For each selection round, all piglets born over a period of 1 week were weighed on the day of birth. Three criteria were used to select LBW piglets: (1) birth weight was a minimum of 1 SD below the litter average, (2) birth weight was a minimum of 1 SD below the study population average, yielding a maximum birth weight of 1050 g, and (3) litter size was a minimum of ten piglets. For each LBW piglet, a NBW piglet was selected from the same litter based on two criteria: (1) piglet had the same sex as the selected LBW piglet, and (2) birth weight was closest to litter average. To improve chances of survival for LBW piglets, non-selected siblings were cross-fostered when litter size exceeded the sow’s number of functional teats. Furthermore, milk replacer was provided when piglets were 2 to 3 days old. One female LBW piglet was euthanized due to complications from a rectal prolapse during the early stages of discrimination training in the judgment bias task. Her data were excluded from analysis, resulting in a final sample size of 41 pigs.

### Housing

At approximately 4 weeks of age, piglets were weaned and moved to the research facility, which was located next to the breeding farm. Per round, pigs were housed in two adjacent pens (measuring approximately 4 × 5 m), with LBW and NBW piglets housed separately. Pigs were separated by birth weight to avoid potential confounding effects of social hierarchy position, as body weight influences a pig’s social rank (O’Connell et al. 2004). Pens had concrete floors and were supplied daily with fresh straw bedding. To protect piglets from the cold, the pens contained a covered piglet nest equipped with rubber mats covered by straw and plasticized PVC slats hanging in front of the entrance. The nests also contained heat lamps until the pigs were approximately 8 weeks old. The research facility was naturally ventilated. Minimum and maximum temperatures ranged from 3 to 34 °C during the first round of the experiment (pigs trained and tested in spring/summer), and from 6 to 30 °C during the second round of the experiment (pigs tested in autumn/winter). Pigs were only tested if they voluntarily entered the testing apparatus, to avoid testing animals in heat stress. Pigs received ^1^/_3_ of their daily food ration in the morning (prior to training) and the remaining ^2^/_3_ in the afternoon (after training). Water was provided ad libitum. Individual recognition of animals was facilitated by a letter sprayed on the pigs’ backs.

### Judgment bias task

#### Apparatus

**Fig. 1:**
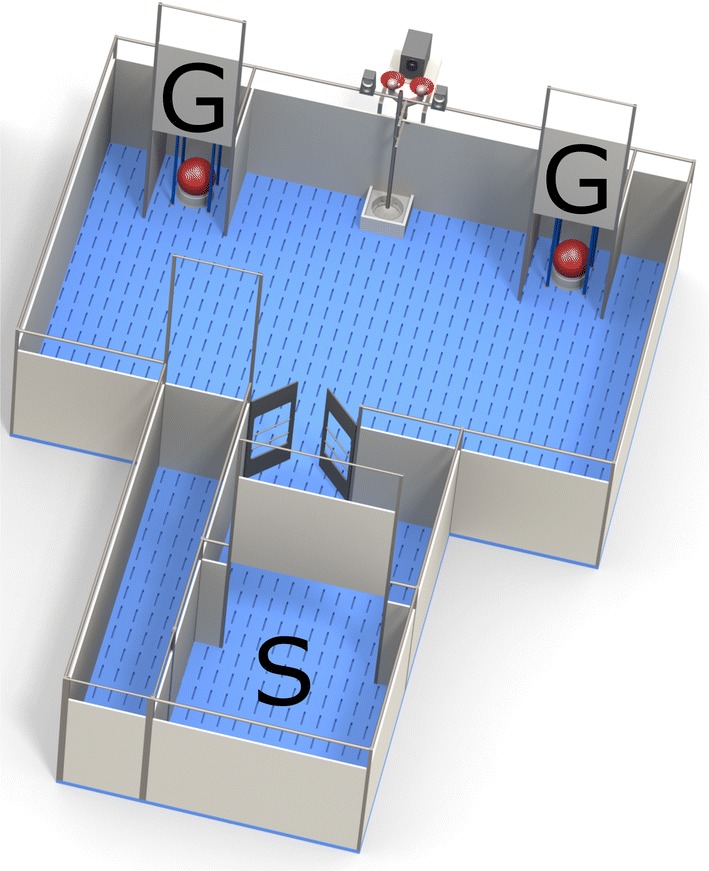
Overview of the judgment bias apparatus, with start box (S) and goal-boxes (G). Each goal-box contains a food bowl covered by a red ball to hide visual cues (illustration: Yorrit van der Staay)

The judgment bias apparatus consisted of a main arena (3.6 × 2.5 m) connected to a start box (1.2 m^2^) by an antechamber (Fig. 1). The start box was equipped with a guillotine door, operated by a rope and pulley system, through which pigs could enter the arena. Two goal-boxes (40 cm wide) were located near the corners at the back of the arena. Each of these contained a food bowl from which the pigs could obtain a reward (M&M’s^®^ Milk Chocolate candies). The food bowls were equipped with a false bottom, beneath which additional candies were placed to avoid discrimination between goal-boxes based on scent cues (for details of food bowls, see Roelofs et al. 2017b). Each food bowl was covered by a synthetic ball (JollyBall Dog Toy, ø 24 cm, 1400 g, Jolly Pets, Ohio, USA), so pigs could not see which bowl contained a food reward. The goal-boxes were equipped with guillotine doors which could be operated from outside the apparatus by rope and pulley systems. Tone-cues were generated using open source software (Audacity; http://audacity.sourceforge.net/) and played by speakers mounted to the back of the arena (Logitech *z*-313, Logitech Europe S.A., Morges, Switzerland). The entire judgment bias apparatus was cleaned daily and rinsed immediately if a pig soiled it during training.

#### Habituation and pre-training

After moving the pigs to the research facility, they were first habituated to being handled by the researchers over a period of 1 week. The pigs were then gradually habituated to the judgment bias apparatus by letting them explore the apparatus in increasingly smaller groups. Habituation finished when all pigs explored the apparatus individually and were able to lift the balls off the food bowls.

Pre-training started with forced trials, which consisted of a pig entering the start box and waiting there for the door to the arena to open. When the door was opened, they could enter the arena and retrieve a single candy from one of the goal-boxes. Only one of the goal-boxes was open and baited per trial, with the location of the reward alternating between the left and right goal-box. After retrieving the reward, the goal-box was closed and the pig returned to the start box for its next trial. Number of trials was gradually increased from six trials until each pig performed a session of 12 consecutive trials.

Next, pigs received four sessions (one session daily) during which they were introduced to a ‘positive’ tone-cue predicting a large reward (four candies) in one goal-box and a ‘negative’ tone-cue predicting a small reward (one candy) in the other goal-box. Two pure tones were used as tone-cues: a 1000 Hz (high) and a 200 Hz (low) tone (waveform: sine, amplitude: 1). The valence of the tone-cues (positive or negative) and the associated goal-boxes (large reward in left or right goal-box) were counterbalanced across animals, for both birth weight and sex. A session of forced trials now consisted of six positive and six negative trials in a pseudorandom order with no more than two identical trials in a row. At the start of a trial, a tone-cue was played while the pig was in the start box. When the pig entered the arena, it could retrieve the appropriate reward from the appropriate goal-box (the other goal-box remained closed). When the pig lifted the ball covering the food bowl to gain access to the reward, the tone-cue was stopped.

The final phase of pre-training consisted of two sessions of ‘open choice’ trials, during which both goal-boxes were open and the pig had to choose a goal-box after hearing the tone-cue. If a pig chose the rewarded goal-box (correct choice), the tone-cue stopped playing, the pig consumed the reward and returned to the start box for its next trial. If a pig chose the unrewarded goal-box (incorrect choice), this goal-box was closed and the tone-cue kept playing until the pig visited the correct goal-box to retrieve the reward. In total, habituation and pre-training took approximately 6 weeks.

#### Discrimination training

Discrimination training consisted of daily sessions of 13 trials each. The first three trials were ‘forced’ trials where only the correct goal-box was open. The first of these forced trials was always a negative trial, followed by a negative and positive trial in a random, daily changing order. Forced trials were followed by five negative and five positive ‘free’ trials in pseudorandom order, with no more than two identical trials in a row. Each daily session had a different order of free trials. Free trials were comparable to open choice trials, except that an incorrect choice was followed by closing both goal-boxes, after which the pig had to return to the start box without receiving a reward. The same consequences applied if a pig did not choose a goal-box within 30 s (recorded as an omission to choose). During every fifth discrimination training session, the first three negative and first three positive free trials were replaced by open choice trials, to allow all pigs to maintain an association between tone-cues and goal-boxes. Discrimination training continued until a pig reached a criterion score of at least four out of five correct choices for both negative and positive free trials for three consecutive training sessions. Pigs that did not reach criterion within a maximum number of 45 training sessions did not proceed to judgment bias testing.

#### Judgment bias testing

For judgment bias testing, each pig performed four sessions, consisting of 16 trials each. A testing session was similar in setup to a training session, with an additional three ambiguous trials. During each of these ambiguous trials, a different ambiguous tone-cue was played. Ambiguous cues were intermediate between the learned positive and negative tones, with frequencies at equal intervals between the training tones on a logarithmic scale: 299.07 Hz, 447.21 Hz and 668.74 Hz. Depending on whether the high or low frequency training tone was used as the positive stimulus, the ambiguous tones represented a ‘near-negative’ ambiguous cue (most similar to the negative tone-cue), an intermediate ambiguous cue (the 447.21 Hz tone) and a ‘near-positive’ ambiguous cue (most similar to the positive tone-cue). For each daily testing session, trials 6, 11 and 16 were the ambiguous trials. The intermediate tone-cue was always presented during trial 6, while the near-negative and near-positive tone-cues alternated between trials 11 and 16. Whether an ambiguous trial was preceded by a negative or positive trial was counterbalanced across testing sessions, to control for potential effects of a preceding trial and its consequences on pigs’ expectations. Ambiguous trials were always unrewarded.

#### Preventing loss of ambiguity

Previous judgment bias studies have shown that leaving ambiguous test trial unrewarded can result in a loss of ambiguity, where animals learn to associate ambiguous stimuli with a lack of reward (Roelofs et al. 2016). Several measures were applied in this study to prevent pigs from learning about the outcome of ambiguous trials. First, from tone introduction onwards, pigs were trained on a partial reinforcement ratio schedule, with an 80% reinforcement ratio as suggested by Düpjan et al. (2017). As a result, one negative and positive trial per session was unrewarded. Unrewarded trials were randomly determined, however, the first and last positive and negative trial of a session was always reinforced. Second, to maintain responsiveness of pigs during unrewarded trials, a secondary reinforcer was used. During training, every correct choice was reinforced with both a clicker and vocal encouragement, as a signal of correct responding even during unrewarded trials. During testing, partial reinforcement of training tones continued, as well as secondary reinforcement of correct responses during non-ambiguous trials. However, during ambiguous trials no reward was present and only vocal encouragement was used when a pig made a choice (i.e., no omission) to avoid providing the pigs with a clear signal of correct or incorrect performance.

#### Variables

The following variables were calculated per pig during discrimination training:

- *Sessions to criterion* was calculated as the number of discrimination training sessions needed to reach criterion level and proceed to judgment bias testing.
- *Number of correct choices* was calculated as the number of correct choices made during a training session, per cue type (excluding forced trials).

The following variables were calculated per pig during judgment bias testing:

- *Optimistic choice* was calculated as the proportion of optimistic choices (i.e., approaching the location associated with a large reward) made during testing sessions, per cue type (negative, positive and ambiguous).
- *Latency to choose* was calculated as average time in seconds elapsed between a pig leaving the start box and lifting a ball in a goal-box, per cue type (negative, positive ,and ambiguous).

### Cortisol analysis

#### Hair cortisol

Hair samples were collected twice: at weaning and at the end of the experiment, when the pigs were approximately 4.5 months old. Hair was collected from the left flank of each pig with a disposable razor (single edged disposable prep razor, Kai Medical, Solingen, Germany), using a new razor for each sample. Determination of hair cortisol concentration was based on a protocol by Davenport et al. (2006). In short, samples were washed and dried after collection. Approximately 35 mg of hair was ground with a bead beater (TissueLyser II, QIAGEN Benelux B.V., Antwerp, Belgium) for a minimum of 2 × 15 min at 30 Hz, in 2 mL tubes containing three 2.3 mm steal beads (BioSpec, Lab Services B.V., Breda, the Netherlands). Corticosteroids were extracted by adding 1 mL methanol to the ground hair and incubating samples for 24 h with slow rotation. Of the extract, 0.6 mL was dried using a vacuum centrifuge. Dried extracts were dissolved by adding 0.3 mL phosphate buffer. Hair cortisol concentrations were then determined in duplo using a Salimetrics Salivary Cortisol ELISA kit. Intra-assay and inter-assay coefficients of variation (CV) were 7.2 and 7.7, respectively.

#### Salivary cortisol

Saliva samples were collected from each pig prior to and after their first individual pre-training session (consisting of six forced trials). Pre-stressor samples were collected at approximately 14:00 in the afternoon in their home pens. Post-stressor samples were taken approximately 20 min after a pig’s pre-training session, to allow for the peak in cortisol response to develop (Merlot et al. 2011). Saliva samples were collected by allowing each pig to chew on two cotton swabs (Cotton Swabs 150 mm × 4 mm WA 2PL; Heinz Herenz, Hamburg, Germany) until they were sufficiently moistened. Saliva was collected from the swabs by centrifuging them in saliva collection tubes (Salivette, Sarstedt, Germany) at around 3524*g* for 10 min at 10 °C. Saliva samples were stored at − 20 °C until salivary cortisol concentration was determined in duplo using a Coat-a-Count radioimmunoassay kit (Siemens Healthcare Diagnostics B.V., The Hague, The Netherlands). Intra-assay and inter-assay CVs were 4.4 and 8.5, respectively.

### Statistical analysis

All statistical analyses were performed using R statistical software, version 3.4.2 (R Core Team 2017). For mixed models, package lme4 (Bates et al. 2015) was used. For linear mixed models the random effect structure was assessed using restricted maximum likelihood (REML) estimation, fixed effect structure was assessed using maximum likelihood (ML) estimation. Model selection was based on Akaike’s information criterion (AIC), using package MuMIn (Barton 2018). Confidence intervals were calculated as 95% parametric bootstrap intervals with 1000 samples. Type III and Wald tests were used to test significance (with *α* = 0.05) of fixed effects of linear models and generalized linear/logistic regression models, respectively. Unless indicated otherwise, results are presented as mean ± SEM.

For mixed models, the following factors were compared as random effects: pig, litter and pen (to account for a potential confounding effect of pen-specific influences). For models used to analyze repeated measures, the additional factors pig nested in litter and pig nested in pen were compared. Round (first or second round of selected animals) did not improve fit of mixed models based on AIC, either as a fixed or random effect, suggesting it did not have explanatory value in the models. Therefore, this factor was not included in analysis.

#### Birth weight and growth

Average birth weight of LBW and NBW pigs was compared using Welch’s *t* test. To compare weekly weight gain of LBW and NBW pigs, a linear mixed model was used with birth weight, week and birth weight × week interaction as fixed effects. Random effect structure consisted of random intercepts for pig nested in litter.

#### Discrimination training

Sessions to criterion for LBW and NBW pigs were compared using a negative binomial generalized linear mixed model to account for overdispersion in the data. Only birth weight was included as a fixed effect, as inclusion of sex did not improve the fit of the model based on AIC. Random effect structure consisted of random intercepts for litter. For number of correct choices during positive and negative training trials, means of three successive sessions (session blocks) were calculated. Performance of LBW and NBW pigs was compared using a logistic regression model with session block as a fixed effect. Inclusion of birth weight, sex or interaction terms did not improve the fit of the model based on AIC. Random effect structure consisted of random intercepts for pig nested in pen. As the total number of training sessions differed per pig, number of correct choices was analyzed for each pig’s first four and last four session blocks (i.e., first and last 12 training sessions).

#### Judgment bias

For optimistic choice and latency to choose, means were calculated across test sessions per cue type. In addition, optimistic choice responses during the first test session were analyzed separately, as these responses would not be confounded by an effect of repeated testing, i.e., loss of ambiguity. Factors assessed during model selection were birth weight, sex, cue type (negative, near-negative ambiguous, intermediate ambiguous, near-positive ambiguous, positive), training duration (discrimination training was completed within 25 sessions or required more than 25 sessions) and all two-way interactions as fixed effects.

Effect of birth weight on optimistic choice was assessed using a logistic regression model. For responses across test sessions, a model with cue type, sex and cue type × sex interaction as fixed effects was most suitable based on AIC. This suggests birth weight was not important to the model. Random effect structure consisted of random intercepts for pen. For responses during the first test session, a model with only cue type as fixed effect was selected, suggesting this to be the only important explaining factor for the data. Random effect structure consisted of random intercepts for litter.

Latency to choose was analyzed using a linear mixed model with cue type and birth weight as fixed effect. Inclusion of additional fixed effects (such as sex) did not improve the fit of the model based on AIC. Random effect structure consisted of random intercepts for pig nested in litter. Latencies to choose were log_10_ transformed to improve distribution of residuals.

#### Loss of ambiguity

To assess whether loss of ambiguity occurred over the course of judgment bias testing, average optimistic choice during the first two test sessions was compared to average optimistic choice during the last two test sessions. A logistic regression model with session (first two sessions combined versus last two sessions combined), cue type and their two-way interaction as fixed effects was used. Random effect structure consisted of random intercepts for pig.

#### Cortisol concentrations

Factors assessed during model selection for hair and salivary cortisol were birth weight, sex and its interaction as fixed effects. For salivary cortisol, an additional factor of sampling time (prior to or post-stressor) was assessed.

The effects of birth weight on pigs’ hair cortisol concentrations were analyzed using a linear mixed model with birth weight, sex and birth weight × sex interaction as fixed effects. Random intercepts for litter were used as random effect structure for hair cortisol concentrations determined at weaning, while random intercepts for pen were most suitable for hair cortisol concentrations determined at the end of the experiment. The effects of birth weight on salivary cortisol concentrations before and after a pig’s first individual pre-training session were analyzed using a linear mixed model with birth weight as fixed effect and random intercepts for pig nested in pen.

To assess a potential relationship between judgment bias and chronic stress, mean optimistic choice percentage (calculated as mean optimistic choice across all four test sessions) in the judgment bias task and mean hair cortisol at the end of the experiment were compared using Spearman’s correlation as hair cortisol concentrations were not normally distributed.

## Results

### Birth weight and growth

**Fig. 2:**
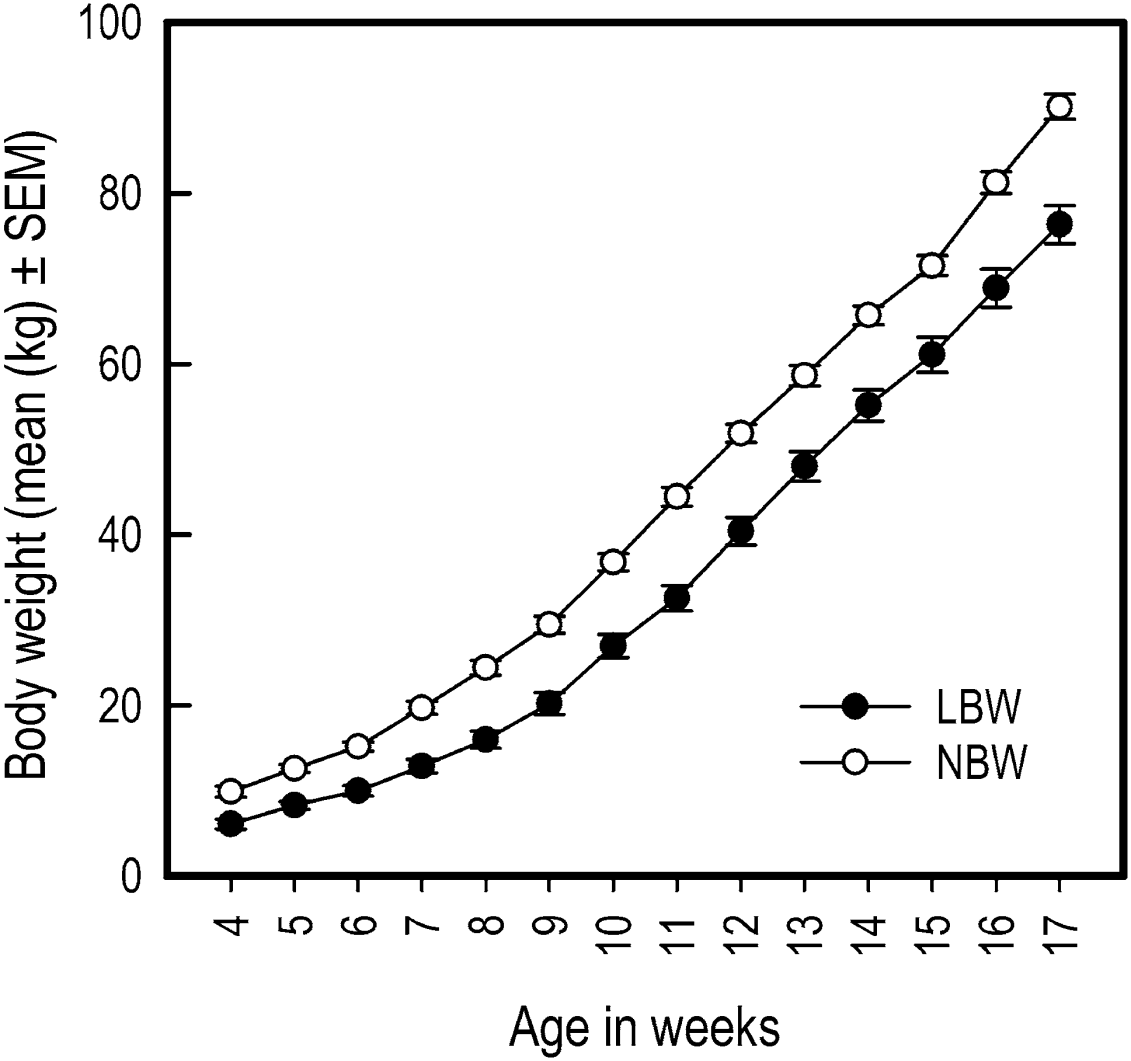
Average body weight in kilograms of LBW and NBW pigs from weaning until the end of the experiment. Error bars are standard errors of the mean

LBW piglets had on average a lower birth weight than NBW piglets (LBW: 0.83 ± 0.10, NBW: 1.47 ± 0.23; *t*_28.45_ = − 11.76, *P* < 0.001, 95% CI [− 0.75, − 0.53]). LBW piglets continued to have lower body weight throughout the duration of the experiment (birth weight: *F*_1,26_ = 95.54, *P* < 0.001, 95% CI [− 7.13, − 1.57]) and had a slower growth rate than the NBW piglets (birth weight × week: *F*_13,487_ = 6.42, *P* < 0.001, 95% CI [− 0.86, − 0.47]; Fig. 2).

### Discrimination training

Of the 41 pigs that started discrimination training, a total of five pigs did not reach criterion performance: two LBW pigs (one female and one male) and three NBW pigs (two females and one male). Of the pigs that completed discrimination training, LBW pigs required a higher number of sessions to reach criterion compared to NBW piglets (LBW: 29.44 ± 8.77, NBW: 24.83 ± 8.89; *X*^2^ = 4.62, *df* = 1, *P* = 0.032, 95% CI [0.02, 0.36]).

**Fig. 3:**
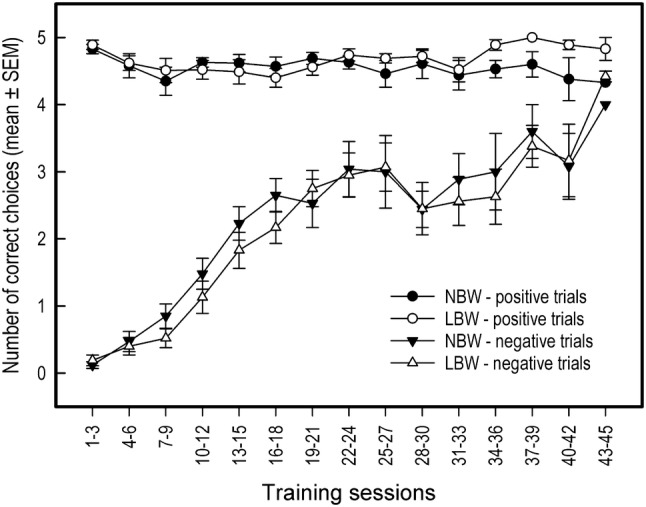
Mean number of correct choices for negative and positive trials during discrimination training of LBW and NBW pigs. Note that from session 14 onwards, individual pigs reached criterion performance and completed discrimination training. Therefore, data shown reflect a gradually decreasing sample size from *n* = 41 at session 14 to *n* = 3 at session 45. Error bars are standard errors of the mean

During discrimination training sessions, the pigs initially only visited the location of the high reward, resulting in very few errors during positive trials, and very few correct choices during negative trials (Fig. 3). On the number of correct choices during training sessions, only session was found to have an effect. As the first twelve training sessions progressed, pigs started to make more correct choices during negative trials (*X*^2^ = 143.86, *df* = 1, *P* < 0.001, 95% CI [0.67, 0.93]; Fig. 3). As the pigs started increasing their visits to the location of the low reward, correct choices during positive trials slightly decreased (*X*^2^ = 20.36, *df* = 1, *P* < 0.001, 95% CI [− 0.47, − 0.18]; Fig. 3). During the last twelve training sessions, the number of correct choices during negative trials was still increasing (*X*^2^ = 213.39, *df* = 1, *P* < 0.001, 95% CI [0.52, 0.68]). Correct responses during positive trials slightly increased for the last twelve training sessions (*X*^2^ = 8.12, *df* = 1, *P* = 0.004, 95% CI [0.06, 0.35]). Note that these analyses were performed on each pig’s final 12 training sessions, with the total number of required training sessions differing per pig. The pigs responded on the majority of discrimination training trials, with omissions occurring on average < 1 trial per training session (LBW: 0.57 ± 1.17 omissions per session, NBW: 0.31 ± 0.89 omissions per session).

### Judgment bias

**Fig. 4:**
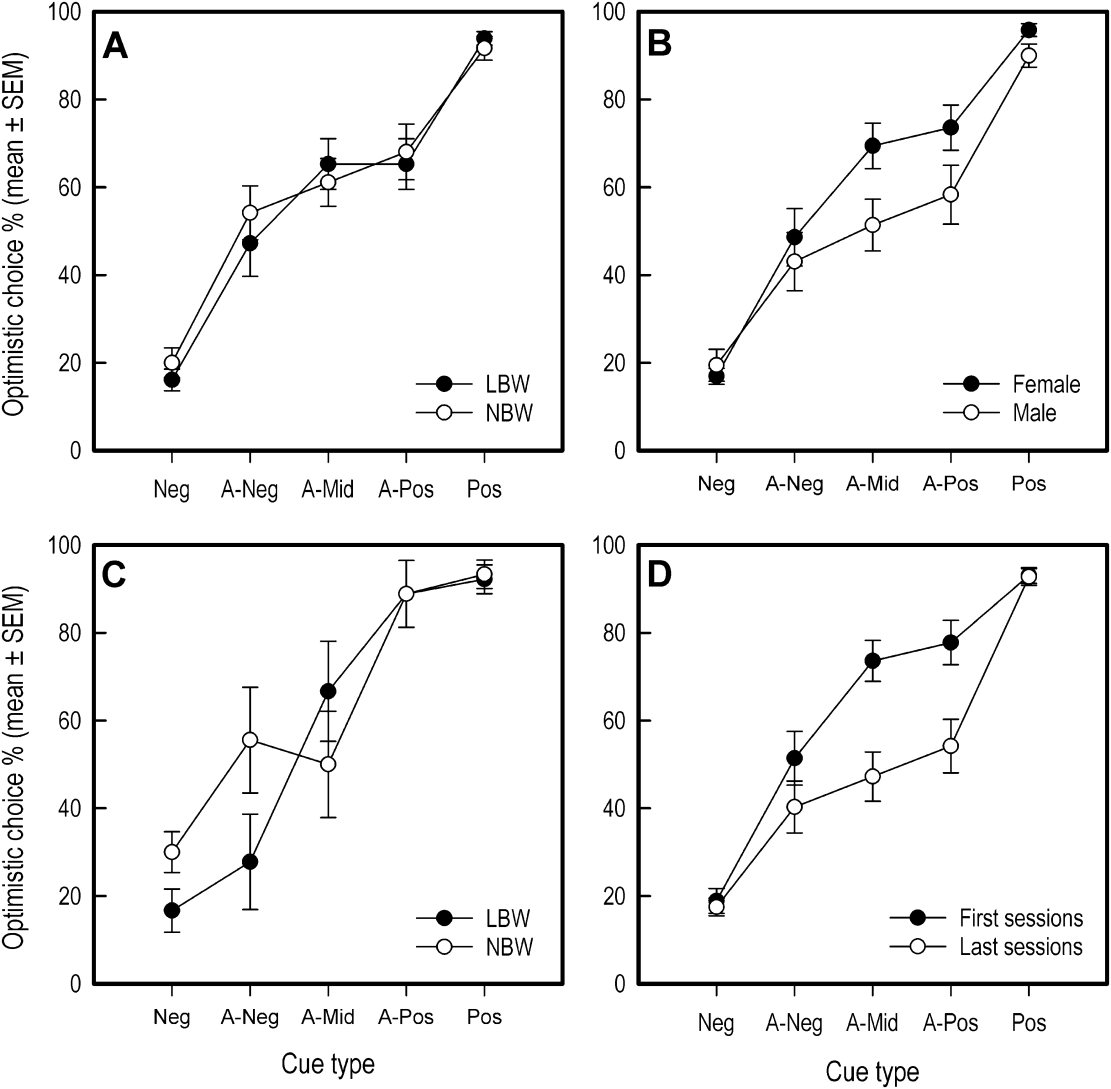
**a** Optimistic choice percentage of LBW and NBW pigs during judgment bias testing (average performance across test sessions). **b** Optimistic choice percentage of female and male pigs during judgment bias testing (average performance across test sessions). **c** Optimistic choice percentage of LBW and NBW pigs during first session of judgment bias testing. As no effect of sex was found, data for female and male pigs are combined. **d** Optimistic choice percentage of all pigs combined for first and last test sessions. Error bars are standard errors of the mean

When analyzing the performance of pigs across all test sessions, the most suitable model included the factors cue type, sex and sex × cue type interaction. All pigs increased their optimistic choices as similarity to the positive tone cue increased (cue type: *X*^2^ = 293.88, *df* = 4, *P* < 0.001, negative vs. positive tone 95% CI [4.18, 5.36]; Fig. 4a). As inclusion of birth weight did not lower model AIC, we can conclude that this factor did not influence optimistic choice during the judgment bias task (Fig. 4a). While no general effect of sex was found (*X*^2^ = 0.74, *df* = 1, *P* = 0.390, 95% CI [− 0.21, 0.55]), the sex × cue type interaction was significant (*X*^2^ = 12.96, *df* = 4, *P* < 0.011; Fig. 4b). This suggests female and male pigs responded differently to certain cue types. Females responded more optimistically to the intermediate ambiguous (*X*^2^ = 4.84, *df* = 1, *P* = 0.027, 95% CI [0.09, 1.47]), near-positive ambiguous (*X*^2^ = 4.66, *df* = 1, *P* = 0.031, 95% CI [0.08, 1.57]) and positive (*X*^2^ = 8.41, *df* = 1, *P* = 0.004, 95% CI [0.32, 1.57]) cues. For pigs’ optimistic choice responses during the first test session, only cue type was found to have an effect (*X*^2^ = 136.56, *df* = 4, *P* < 0.001, negative vs. positive tone 95% CI [3.19, 4.57]; Fig. 4c).

**Fig. 5:**
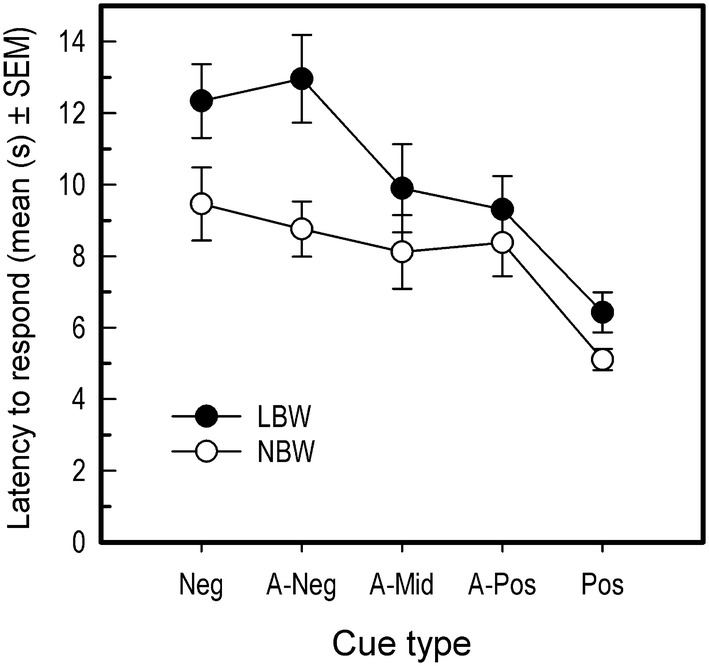
Average latency to respond during testing trials of LBW and NBW pigs. As no effect of sex was found, data for female and male pigs are combined. Error bars are standard errors of the mean

For latency to choose, the model containing cue type and birth weight as fixed effects was most suitable based on AIC. Latency to choose decreased as similarity to the positive tone cue increased (*F*_4, 140_ = 32.24, *P* < 0.001, negative vs. positive tone 95% CI of log_10_ transformed latencies [− 0.31, − 0.21]; Fig. 5). Furthermore, LBW pigs had slightly higher latencies to respond than NBW pigs (*F*_1, 21_ = 5.99, *P* < 0.023; 95% CI of log_10_ transformed latencies [0.02, 0.18]; Fig. 5).

Similar to training sessions, the occurrence of omissions during testing sessions was low. Omissions occurred on average < 1 trial per testing session (LBW: 0.03 ± 0.04 omissions per session, NBW: 0.01 ± 0.02 omissions per session).

### Loss of ambiguity

When assessing potential loss of ambiguity due to repeated testing by comparing average optimistic choice of pigs during the first versus the last two test sessions, no general effect of session was found (*X*^2^ = 0.24, *df* = 1, *P* = 0.627, 95% CI [− 0.48, 0.29]). However, the session × cue type interaction did affect optimistic choice (*X*^2^ = 12.00, *df* = 4, *P* < 0.017), implying pigs changed their responses to certain tone cues over the course of testing. This was confirmed for the intermediate (*X*^2^ = 9.89, *df* = 1, *P* = 0.002, 95% CI [− 1.93, − 0.46]) and near-positive (*X*^2^ = 8.87, *df* = 1, *P* = 0.003, 95% CI [− 2.01, − 0.43]) ambiguous cues, with pigs reducing their optimistic choice as testing sessions progressed (Fig. 4d).

### Cortisol concentrations

#### Hair cortisol

No effects of birth weight were found on hair cortisol concentrations, either at weaning (*F*_1,24_ = 2.16, *P* = 0.155, 95% CI [− 9.43, 0.37]) or at the end of the experiment, at 5 months of age (*F*_1,2_ = 0.00, *P* = 0.991, 95% CI [− 15.92, 15.40]). Sex did not influence hair cortisol concentrations, neither as a main effect (at weaning: *F*_1,24_ = 0.00, *P* = 0.980, 95% CI [− 7.54, 3.68]; at 5 months old: *F*_1,35_ = 0.64, *P* = 0.429, 95% CI [− 4.74, 7.43]), nor in interaction with birth weight (at weaning: *F*_1,24_ = 1.25, *P* = 0.275, 95% CI [− 3.01, 10.83]; at 5 months old: *F*_1,35_ = 0.04, *P* = 0.850, 95% CI [− 7.66, 9.37]).

No correlation was found between hair cortisol concentrations at the end of the experiment and mean optimistic choice percentage in the judgment bias task (*r*_s_ = − 0.06, *P* = 0.73).

#### Salivary cortisol

As sampling time did not influence salivary cortisol concentrations, we can conclude that the pigs did not show an acute stress response to their first individual training session. Although the model with birth weight as fixed effect had the lowest AIC, no difference between LBW and NBW pigs was found (*F*_1,2_ = 0.13, *P* = 0.754, 95% CI [− 1.90, 1.41]).

## Discussion

In the present study we assessed the effects of low birth weight (LBW) and sex on discrimination learning and judgment bias in pigs. We were successful in selecting piglets with a significantly lower birth weight than the selected normal birth weight (NBW) pigs. This is important, as LBW is currently the best read-out parameter of intra-uterine growth restriction and its associated effects on brain development in pigs (Wu et al. 2006; Gieling 2013). Based on previous studies with humans and pigs, we expected to find both a cognitive impairment (O’Keeffe et al. 2003; Gieling et al. 2012; Radlowski et al. 2014; Yu and Garcy 2018; Roelofs et al. 2018) and a more negative judgment bias (Lahti et al. 2010; Murphy et al. 2015) in LBW pigs. LBW pigs were slower to consistently perform the correct behaviors in response to the correct cues. However, no differences between LBW and NBW pigs were found in judgment bias. This finding of a similar emotional state in both groups of pigs is supported by similar hair cortisol concentrations, which were used as a marker for chronic stress.

### Discrimination learning

Birth weight had a mild effect on pigs’ cognitive abilities, based on the number of discrimination training sessions required to reach criterion performance. LBW pigs took longer to finish discrimination training compared to NBW pigs. This finding indicates that the LBW pigs had more difficulty learning the rules of the task, i.e., they took longer to consistently perform the correct responses to the training cues. Such a cognitive impairment was expected based on the substantial difference in birth weight between groups, as well as the lack of catch-up growth displayed by the LBW pigs. Catch-up growth has been shown to limit the risks for cognitive impairment in humans (Lindström et al. 2017). It is unlikely that this difference in performance between LBW and NBW pigs was due to a difference in motivation to perform the task. Both groups of pigs showed comparable latencies to respond to training cues during judgment bias testing. Furthermore, LBW and NBW pigs do not differ in their food motivation in operant conditioning tasks (van Eck et al. 2016).

Our finding of a post-weaning cognitive impairment in LBW pigs is comparable to results of multiple human studies. These studies also show impaired cognitive performance in LBW children which persists well beyond childhood (e.g., Kormos et al. 2014; Lindström et al. 2017). In humans, the negative cognitive effects of LBW can be limited by ameliorating circumstances such as positive family attitudes (Yu and Garcy 2018). Perhaps the enriched housing conditions applied during our study also had such an ameliorating effect on the pigs’performance (Sneddon et al. 2000; Grimberg-Henrici et al. 2016), resulting in only a mild impairment compared to the NBW pigs. Similar mild effects have also been found for other cognitive domains in LBW pigs, such as spatial learning and memory (Gieling et al. 2012; Radlowski et al. 2014; Roelofs et al. 2018; cf. Antonides et al. 2015). However, the only other studies comparing LBW and NBW pigs in a discrimination training task found no effects of birth weight, with both groups of pigs requiring a similar number of training sessions to complete the task (Murphy et al. 2013a, 2015).

The difficulty in the discrimination training task lies in correct responding to the negative cue. Pigs show a strong preference for the larger reward available after presentation of the positive cue, causing them to respond incorrectly during negative trials. During the first training sessions, pigs almost exclusively approach the positive goal-box, irrespective of the presented tone-cue. This has also been found in previous discrimination training tasks with pigs (e.g., Murphy et al. 2013a), as well as in a delay discounting task (Melotti et al. 2013). In the delay discounting task, the pigs’ preference for a larger reward was so strong that instead of performing a response leading to a small reward, they resorted to omissions and failed to gain a reward at all.

The training protocol used in our study could have led to a more difficult cognitive challenge for the pigs, compared to the protocol used in the studies by Murphy et al. (2013a, 2015). In those studies, continuous reinforcement was applied during discrimination training, where a correct response (i.e., approaching the correct goal-box) was always rewarded. In our study, we applied a partial reinforcement schedule with one out of six training trials going unrewarded, irrespective of a pig’s response. A possible difference in task difficulty between our study and those of Murphy et al. is reflected by the difference in required training sessions until the pigs reach criterion performance. In our study, pigs required an average of 25–30 training sessions, compared to an average of 16 training sessions needed in the study by Murphy et al. (2015).

In theory, both our study and those by Murphy et al. (2013a, 2015) applied a differential outcome paradigm (Holden and Overmier 2014). In such a paradigm, the two types of stimulus–response sequences (positive cue followed by approach to positive goal-box and negative cue followed by approach to negative goal-box) are followed by specific, different rewards (i.e., different quantities of food reward). However, it is possible the LBW pigs in our study had more difficulty reaching criterion performance because correct responses were not consistently rewarded. Task acquisition in general has been shown to slow down when partial reinforcement is applied (Sangha et al. 2002; Grady et al. 2016), especially when training sessions are widely spaced, as was the case in our study with one training session per day (Robbins 1971). In addition, the unrewarded trials during each session had non-differential outcomes (i.e., both stimulus–response sequences lead to the same outcome of no reward; Holden and Overmier 2014). A negative effect of non-differential outcomes has also been shown in LBW children, who only reach comparable performance to NBW children when trained on a differential outcome paradigm (Martínez et al. 2012).

### Judgment bias

We found no effect of birth weight on judgment bias. LBW and NBW pigs displayed similar rates of optimistic choice in response to ambiguous tone-cues. This was found for all testing sessions combined and when the first testing session was analyzed separately, to ensure results were not confounded by loss of ambiguity. These findings suggest that the LBW pigs in our study did not suffer from a more negative emotional state than the NBW pigs. We did find a difference in latency to respond between LBW and NBW pigs, with LBW pigs taking slightly longer to respond after being presented with a tone cue. However, considering the similar rates of optimistic choice for LBW and NBW pigs, it is unlikely the difference in latency to respond reflects a difference between groups in emotional state. Rather, it is probable that this measure was confounded by the difference in body size and muscle strength between LBW and NBW pigs. Pigs’ stride length increases with body size (Stavrakakis et al. 2014). Additionally, LBW pigs have impaired muscle development compared to NBW pigs (Beaulieu et al. 2010; Berard et al. 2010), potentially affecting their locomotion. A lack of difference in emotional state between LBW and NBW pigs was also suggested by the similar hair and salivary cortisol concentrations found. Human studies have reported an increased risk for emotional disorders such as anxiety and depression in LBW children (Lahti et al. 2010; Boyle et al. 2011; Lahat et al. 2017). Similarly, LBW pigs in a previous study showed a more pessimistic judgment bias in response to ambiguous stimuli than NBW pigs (Murphy et al. 2015). It is possible that we did not find a more negative emotional state in LBW pigs because they were housed in enriched conditions, which are known to have a positive effect on emotional state (de Jong et al. 2000; Douglas et al. 2012). In pigs, LBW has been shown to lead to increased vulnerability to stressors (Poore and Fowden 2003) and in humans this vulnerability is more pronounced in females (Van Lieshout and Boylan 2010). However, based on our housing conditions and the results from hair and salivary cortisol analysis, neither the LBW nor the NBW pigs were stressed. This lack of stress could explain why we were unable to detect (sex-specific) effects of LBW on emotional state. Furthermore, several differences in study design could have contributed to the discrepancy in results between our study and that performed by Murphy et al. (2015).

First, our study used a bigger sample size of 20 LBW versus 20 NBW pigs, compared to eight LBW versus eight NBW pigs tested by Murphy et al. (2015). Using a smaller sample size not only increases the chance of false negative results (because of low power), but it also increases the risk of chance findings (Button et al. 2013). That is, studies with smaller sample sizes are at greater risk of finding a significant result due to factors other than the assumed effect. In pigs, baseline emotional state can vary between individuals, even those experiencing similar (environmental) conditions. For example, differences in personality have been shown to affect baseline emotional responding in pigs (Krause et al. 2017). The notion that individual differences within a treatment group could be a confounding factor when assessing emotional state is supported by the high in variability shown our study. Within the LBW and NBW groups, there were considerable differences in optimistic choice percentage, with certain animals being more optimistic than others irrelative of birth weight. A study based on a smaller sample size could therefore lead to statistically significant findings that do not reflect a true effect, if by coincidence a subset of more pessimistic LBW pigs and/or a subset of more optimistic NBW pigs were chosen.

Besides a difference in sample size, it is possible that the LBW pigs tested by Murphy et al. (2015) were more stressed than those tested in our study due to differences in social environment. First, their LBW and NBW pigs were housed in mixed groups, whereas the pigs in our study were housed separately per birth weight category. A pig’s social rank and body weight are correlated, with larger pigs often having a higher position in the dominance hierarchy (Litten et al. 2003; O’Connell et al. 2004). As LBW pigs remain smaller than their NBW siblings, it is likely that when they are housed together, NBW pigs will have a higher social rank than the LBW pigs. This could have resulted in a more negative emotional state for the LBW pigs tested by Murphy et al. (2015). Such an effect has previously been found in rats, with lower ranking female rats showing a more negative judgment bias than those with a higher social rank (Barker et al. 2018). In pigs, only indirect measures of emotional state have been assessed in correlation with their social status. These studies also suggest that in stable hierarchies, lower ranking pigs may have a more negative emotional state, as they have more injuries, lose competition over food and display more fearful behaviors in a novel object test (O’Connell et al. 2004; Boumans et al. 2018). It is important to note that in our study, avoiding confounding effects of social rank by separate housing of pigs by treatment group may have resulted in other potential confounds. Any chance differences in (social) environment between pens may have influenced our results, independently of birth weight. However, such potential confounds were limited by performing the experiment in multiple rounds, as well as exploring potential effects of pen environment in statistical analysis.

Second, Murphy et al. (2015) tested only male pigs, whereas the current study included both sexes, housed in mixed-sex groups. Group composition may affect the emotional state of pigs, as males and females may have different social behaviors. For example, male pigs are reported to initiate more aggressive interactions (Clark and D’Eath 2013; Puls et al. 2017), and to perform higher rates of mounting behaviors than females (Clark and D’Eath 2013; Puls et al. 2017). Mounting behaviors are a likely cause of stress, as recipients produce high-pitched screams (Hintze et al. 2013). While these studies suggest males in single-sex groups may be more stressed, it has also been reported that male pigs behave more aggressively when housed in mixed-sex groups as compared to single-sex housing (Colson et al. 2006). Further indication that group composition may influence emotional state in pigs comes from a finding of impaired behavioral flexibility in male pigs when housed in a single-sex group (Roelofs et al. 2017b). When this study was repeated with mixed-sex housing, no difference in behavioral flexibility between females and males was found (Roelofs et al. 2018). The composite effects of various elements of pigs’ social environment are likely complex, as is also indicated by our finding of male pigs responding more pessimistically than females. In previous judgment bias studies, no effects of sex on baseline judgment bias in pigs was found (mixed-sex groups: Carreras et al. 2016; Asher et al. 2016; single-sex groups: Roelofs et al. 2017b).

It is unlikely that higher number of discrimination training sessions applied in our study influenced judgment bias, as no effect of training duration on optimistic choice percentage was found. This is corroborated by a previous study showing that the number of discrimination training sessions is independent from optimistic choosing in the judgment bias task (Roelofs et al. 2017a). Overall, it appears that differences in social environment between our study and the study by Murphy et al. (2015) influenced the emotional state of the pigs, either through a difference in social rank of the LBW pigs or a difference in group composition. This makes it difficult to directly compare the results of these studies. Future studies assessing the emotional state of pigs with different positions in the dominance hierarchy and comparing mixed- and single-sex housing are encouraged.

### Loss of ambiguity

Multiple judgment bias studies have reported a loss of ambiguity due to repeated testing (e.g., Doyle et al. 2010; Karagiannis et al. 2015). Loss of ambiguity can occur when animals learn about the outcome of ambiguous trials, i.e., trials during which they are presented with an ambiguous stimulus (Roelofs et al. 2016). Most often in judgment bias tasks, ambiguous trials go unrewarded while during training, rewards were always present after a correct response. As a result, the lack of reward during ambiguous trials stands out, facilitating learning about the outcome of such trials (Jamieson et al. 2012). Once animals learn to associate ambiguous trials with a specific outcome (i.e., absence or presence of reward), they could adjust their responses accordingly (e.g., display pessimistic responses or omissions when they know no reward is available). Thereby, loss of ambiguity can influence results of judgment bias tasks (Doyle et al. 2010). Such effects of loss of ambiguity have also been reported for pigs (Murphy et al. 2013b; Scollo et al. 2014; Roelofs et al. 2017b). To prevent loss of ambiguity, partial reinforcement schedules have been successfully applied during discrimination training (e.g., Bateson et al. 2015; Bethell and Koyama 2015; Düpjan et al. 2017). In these studies, a pre-defined percentage of training trials goes unrewarded, increasing similarity between training and testing conditions.

In our study, applying partial reinforcement of training trials was not successful as a means of avoiding loss of ambiguity. Both LBW and NBW pigs decreased their optimistic choice percentage as judgment bias testing sessions progressed. The pigs did not switch from approaching a goal-box to omissions, as might be expected when no reward is available. Such a shift to omissions has previously been reported for starlings which were repeatedly tested in a judgment bias task (Brilot et al. 2010). As omissions occurred only sporadically in the present study, the observed decrease in optimistic responding was the result of pigs switching to pessimistic responses during ambiguous trials. A similar effect of repeated testing was observed in another study assessing judgment bias in pigs (Murphy et al. 2013b). Pigs were trained and tested over three consecutive phases in the same judgment bias task as applied in the present study. As judgment bias testing progressed, pigs initially switched from optimistic to pessimistic responses. Only during the last phase of testing did pigs switch to omissions. Such an initial shift from optimistic to pessimistic responding is likely due to lack of reward being a signal of an incorrect choice during training trials. Based on the study by Murphy et al. (2013b), only after pessimistic responses also fail to result in a reward will pigs cease to make an approach altogether.

The present study’s results suggest the outcome of ambiguous trials still stood out to the pigs, in spite of their experience with unrewarded trials during discrimination training. Perhaps this would not have been the case if the ratio of reference trials to ambiguous trials had been higher. Currently, three out of 16 testing trials were ambiguous and unrewarded. If the ambiguous trials are less frequent, the pigs are provided with less opportunity to learn about their outcome. A recent study successfully applied a study design where testing sessions consisted of 50 reference trials and three ambiguous trials (Hintze et al. 2018). To speed up the process of training and testing a sufficient number of animals on such a considerable number of daily trials, a task was used where animals could ‘opt out’ of negative trials by initiating a new trial. This way, the long latencies to respond during negative trials are avoided. Validation of such a judgment bias task for pigs could potentially increase the reliability of results by avoiding loss of ambiguity.

## Conclusions

Our results show that LBW causes a mild impairment of conditional discrimination learning in pigs. No effects of birth weight on judgment bias were found, suggesting that LBW pigs do not necessarily have a more negative emotional state than NBW pigs. This finding was supported by similar hair and salivary cortisol concentrations for LBW and NBW pigs. It is likely that the enriched housing conditions applied during our study (and the segregation of pigs by size, potentially affecting social structure) contributed to these findings. Finally, the use of partial reinforcement during discrimination training was unsuccessful in avoiding loss of ambiguity during judgment bias testing. Further improvement of judgment bias task designs for pigs are therefore encouraged.
